# DeepMVP: deep learning models trained on high-quality data accurately predict PTM sites and variant-induced alterations

**DOI:** 10.1038/s41592-025-02797-x

**Published:** 2025-08-26

**Authors:** Bo Wen, Chenwei Wang, Kai Li, Ping Han, Matthew V. Holt, Sara R. Savage, Jonathan T. Lei, Yongchao Dou, Zhiao Shi, Yi Li, Bing Zhang

**Affiliations:** 1 Lester and Sue Smith Breast Center, Baylor College of Medicine, Houston, TX USA; 2 Department of Molecular and Human Genetics, Baylor College of Medicine, Houston, TX USA; 3 Department of Molecular and Cellular Biology, Baylor College of Medicine, Houston, TX USA; 4 Present Address: Department of Genome Sciences, University of Washington, Seattle, WA USA; 5 Present Address: Department of Computational Medicine and Bioinformatics, University of Michigan, Ann Arbor, MI USA

**Keywords:** Proteome informatics, Machine learning, Post-translational modifications, Genomics, Proteomics

## Abstract

Post-translational modifications (PTMs) are critical regulators of protein function, and their disruption is a key mechanism by which missense variants contribute to disease. Accurate PTM site prediction using deep learning can help identify PTM-altering variants, but progress has been limited by the lack of large, high-quality training datasets. Here, we introduce PTMAtlas, a curated compendium of 397,524 PTM sites generated through systematic reprocessing of 241 public mass-spectrometry datasets, and DeepMVP, a deep learning framework trained on PTMAtlas to predict PTM sites for phosphorylation, acetylation, methylation, sumoylation, ubiquitination and N-glycosylation. DeepMVP substantially outperforms existing tools across all six PTM types. Its application to predicting PTM-altering missense variants shows strong concordance with experimental results, validated using literature-curated variants and cancer proteogenomic datasets. Together, PTMAtlas and DeepMVP provide a robust platform for PTM research and a scalable framework for assessing the functional consequences of coding variants through the lens of PTMs.

## Abstract (web summary)

DeepMVP is a deep learning framework for predicting PTM sites and variant-induced alterations across six modification types, including phosphorylation, acetylation, methylation, sumoylation, ubiquitination and N-glycosylation.

## Main

Post-translational modifications (PTMs) are critical regulators of protein activity, stability, localization and interactions, playing crucial roles in cellular signaling, metabolism, response to environmental stimuli and many other processes ^1^. PTM disruption is increasingly recognized as a mechanism by which missense variants contribute to diseases^2–8^. Missense variants can affect PTMs in several ways. First, a variant at a PTM site may abolish the PTM by altering the specific residue subjected to the modification. Second, a variation can introduce a new PTM site at the variant location, such as changing a non-serine, non-threonine or non-tyrosine residue to S, T or Y for phosphorylation. Moreover, a variant in the vicinity of a PTM site might influence the likelihood of the modification occurring at that site.

Computational prediction of PTM sites can help identify PTM-altering variants^9,10^. For example, MIMP estimates the impact of missense variants on phosphorylation by using position weight matrices and Gaussian mixture models to compare the predicted kinase-binding probabilities of wild type (WT) and mutant sequences^9^. However, these tools are designed specifically for phosphorylation and operate in a kinase-specific manner, limiting their applicability to well-characterized kinases with sufficient known substrates and excluding less-studied kinases and other types of PTMs. More recently, deep-learning-based, enzyme-agnostic models have been developed for predicting PTM sites across multiple PTM types^11^, expanding the potential for variant prediction beyond well-characterized kinases and phosphorylation. However, the accuracy of these models—essential for reliably assessing the effects of variants on PTMs—remains limited, partly owing to the scarcity of large, high-quality training datasets.

Existing deep learning models for PTM site prediction rely on PTM sites available from public databases for training^11,12^. These databases compile PTM sites from individual studies, identified primarily through mass spectrometry (MS)-based shotgun proteomics, without applying global quality control (QC) measures. Different studies use different protein identifiers and QC criteria, complicating data standardization and integration. As an example, using different protein databases for shotgun proteomics searches can result in different mapping positions for the same PTM sites due to differences in protein sequences across these databases. More importantly, although true identifications across studies often overlap, false identifications are generally random. Consequently, the naive aggregation of PTM sites from studies individually controlled for a 1% false discovery rate (FDR) can lead to a substantially higher global FDR in databases encompassing numerous studies. It has been reported that 55% of phosphosites in PhosphoSitePlus (PSP)^13^, the most widely used PTM database, are supported by only a single piece of MS/MS evidence; this figure was reduced to 11.5% when controlling the global FDR at 1% (ref. ^14^). We reason that high-quality training data generated through systematic reanalysis of public MS proteomics datasets, combined with effective deep learning algorithms, could substantially improve PTM site prediction and enable reliable assessment of variant effects on PTMs.

In this study, we systematically reanalyzed 241 PTM-enriched tandem mass spectrometry (MS/MS) datasets to generate a high-confidence training set of 397,524 PTM sites spanning six major PTMs: phosphorylation, acetylation, ubiquitination, sumoylation, methylation and N-glycosylation. We compiled these sites into PTMAtlas and used them to train DeepMVP (deep learning-based post-translational modification and variant-induced alteration prediction), a deep learning framework for predicting PTM sites and variant-induced alterations across the six modification types. DeepMVP outperformed existing models in PTM site prediction and enabled proteome-wide identification of PTM sites across both human and viral proteomes. To evaluate DeepMVP’s ability to identify PTM-altering variants, we tested it on a manually curated set of literature-derived variants and two cancer proteogenomic datasets, observing strong alignment with experimental evidence. As an exploratory application, we applied DeepMVP to all pathogenic germline variants cataloged in ClinVar^15^ and somatic mutations from a pan-cancer study by The Cancer Genome Atlas (TCGA) network^16^ to identify candidates that might alter PTM sites. The interpretability of DeepMVP further enabled preliminary linkage of predicted PTM changes to potential modifying enzymes, offering a basis for future studies of regulatory mechanisms and therapeutic hypotheses. PTMAtlas, DeepMVP and a Python package for seamless integration of DeepMVP into genomics pipelines are available at http://deepmvp.ptmax.org.

## Results

### An overview of the study

**Fig. 1:**
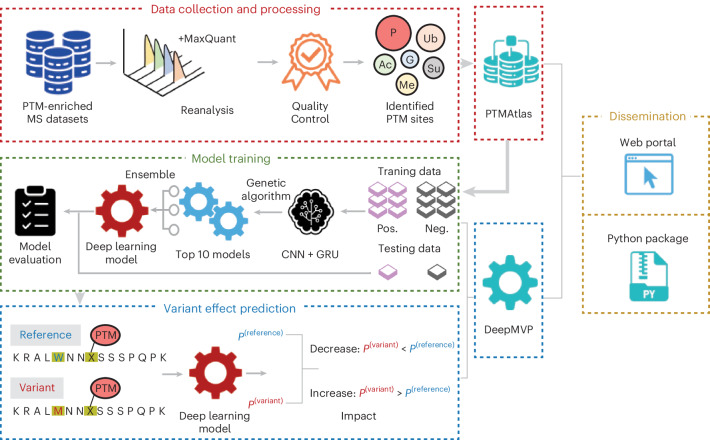
Study design overview. The study comprised several key steps: (1) collection and analysis of published PTM-enriched MS/MS datasets covering six PTM types (phosphorylation (P), acetylation (Ac), methylation (Me), sumoylation (Su), ubiquitination (Ub) and N-glycosylation (G)) to create a comprehensive, high-quality PTM site database known as PTMAtlas. (2) Development of DeepMVP deep learning models for PTM site prediction using data from PTMAtlas. (3) Quantification of the variant-induced effects on PTMs by applying DeepMVP to predict PTM probabilities for both reference and variant protein sequences, followed by calculating the difference in predicted probabilities. (4) Distribution of DeepMVP and PTMAtlas, available through a web portal and a Python package. *P*(variant), the probability of PTM occurrence at the specific site in the variant sequence; *P*(reference), the probability at the specific site in the reference sequence.

Our study involved collecting and processing PTM datasets, training deep learning models to predict PTM sites, applying these models to assess variant effects and disseminating the resulting resources (Fig. 1). We began by reanalyzing raw PTM-enriched MS/MS data from public repositories, focusing on phosphorylation, acetylation, methylation, sumoylation, ubiquitination and N-glycosylation. Using a standardized protocol with strict QC, we generated a high-confidence set of PTM site identifications, compiled into PTMAtlas.

On the basis of PTMAtlas, we developed DeepMVP, a suite of enzyme-agnostic models tailored to each PTM type. Enzyme-agnostic modeling was prioritized owing to the limited availability of known substrates for most modifying enzymes. Model architectures, incorporating both convolutional neural networks (CNNs) and bidirectional gated recurrent units (GRUs), were optimized using a genetic algorithm, and robustness was enhanced through model ensembling.

To assess variant effects, DeepMVP computes PTM probabilities for both reference and variant protein sequences and calculates a delta score on the basis of the difference in predicted modification probabilities at the candidate PTM site. A positive delta score indicates an increased likelihood of PTM occurrence, whereas a negative one suggests a decrease. The PTM site can be located at the variant position (direct effect) or nearby (proximal effect). For direct effects, the delta score is equivalent to the predicted probability of the modifiable residue—either the reference (in cases of loss) or the variant (in cases of gain)—because the non-modifiable counterpart has an inherent probability of zero. Both PTMAtlas and DeepMVP are publicly available.

### PTMAtlas: a comprehensive, high-quality PTM site database

**Fig. 2:**
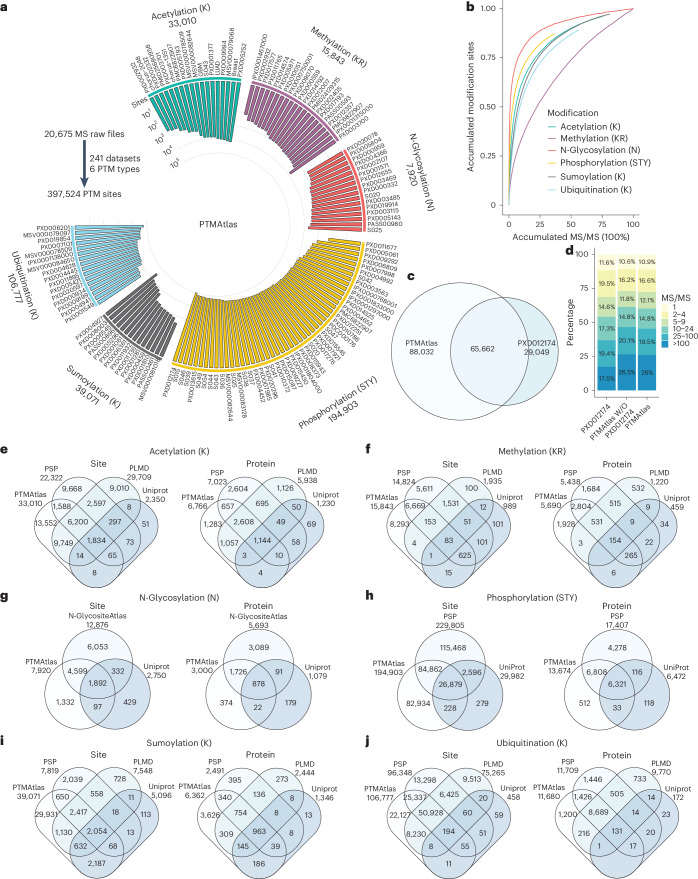
Creation of PTMAtlas and comparison with other PTM databases. **a**, Distribution of collected PTM-enriched MS/MS datasets and the identified PTM sites across the six PTM types. **b**, Rarefaction curve analysis for the six PTM types. **c**, Comparison of phosphorylation sites identified in PTMAtlas and PXD012174. **d**, Comparison of the count distribution of supporting PSMs for all phosphorylation sites identified in PXD012174, all phosphorylation sites identified in PTMAtlas and phosphorylation sites identified in PTMAtlas but not PXD012174. **e**–**j**, Comparison of PTMAtlas and other databases in terms of the number of identified PTM sites and associated proteins across six PTM types.

**Extended Data Fig. 1:**
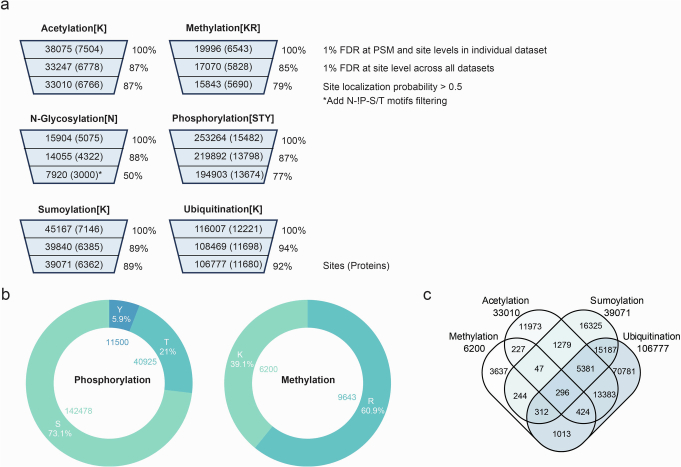
Detailed information regarding PTM site identification in PTMAtlas. **a** Multi-step filtering process for PTM site identification, including dataset-specific PSM and site-level FDR control, cross-datasets site-level FDR control, and site localization probability filtering. Numbers in the parentheses represent corresponding protein numbers. **b** Distribution of modified amino acids for phosphorylation and methylation. **c** Substrate overlap for the four types of PTMs targeting lysine.

We collected 241 human PTM-enriched MS/MS datasets from public repositories, covering six major PTM types: acetylation (lysine, K), methylation (lysine and arginine, K/R), N-glycosylation (asparagine, N), phosphorylation (STY), sumoylation (K) and ubiquitination (K), totaling 20,675 raw files (Fig. 2a and Supplementary Table 1). These included 18 datasets for acetylation (1,122 raw files), 20 for methylation (680 raw files), 16 for N-glycosylation (2,126 raw files), 157 for phosphorylation (15,329 raw files), 14 for sumoylation (850 raw files) and 16 for ubiquitination (568 raw files). Notably, 110 phosphorylation datasets were sourced from a large-scale meta-analysis project (PXD012174)^14^. All raw files were reanalyzed using MaxQuant^17^ (Methods), and the FDRs^18^ were computed at both the peptide–spectrum match (PSM) and PTM site levels. We retained PTM sites that met a 1% FDR threshold at both levels, enforced within and across datasets for each PTM type. PTM sites with a localization probability below 0.5 were excluded (Extended Data Fig. 1a).

We identified a total of 397,524 PTM sites across the six PTM types, including 33,010 acetylation sites on 6,766 proteins, 15,843 methylation sites on 5,690 proteins, 7,920 N-glycosylation sites on 3,000 proteins, 194,903 phosphorylation sites on 13,674 proteins, 39,071 sumoylation sites on 6,362 proteins and 106,777 ubiquitination sites on 11,680 proteins (Fig. 2a and Extended Data Fig. 1a). Rarefaction curve analysis indicated that saturation had not been reached for any PTM type (Fig. 2b). For N-glycosylation, the addition of new datasets—particularly those using similar MS/MS methods—is less likely to yield new sites, whereas methylation datasets are more likely to contribute new identifications. Among the 194,903 phosphorylation sites, 73.1% were on serine, 21% on threonine and 5.9% on tyrosine (Extended Data Fig. 1b). For methylation, 39.1% of the 15,843 sites were on lysine and 60.9% on arginine (Extended Data Fig. 1b). Notably, among the four lysine-targeting PTMs, we identified 296 sites susceptible to all four modification types (Extended Data Fig. 1c and Supplementary Table 2).

We integrated all identified PTM sites into PTMAtlas. To evaluate the quality of these identifications, we compared the phosphorylation sites identified by PTMAtlas in PXD012174 with those reported in the original publication^14^. PTMAtlas recovered 69% of the original PXD012174 sites, whereas the original dataset covered only 43% of the PTMAtlas sites (Fig. 2c). The proportion of sites supported by a single PSM was comparable between all PTMAtlas sites (10.9%) and PTMAtlas-specific sites (10.6%), similar to the 11.6% reported in the original PXD012174 set (Fig. 2d). Notably, 26% of PTMAtlas sites were supported by more than 100 PSMs, compared with 17.5% in the original collection.

**Extended Data Fig. 2:**
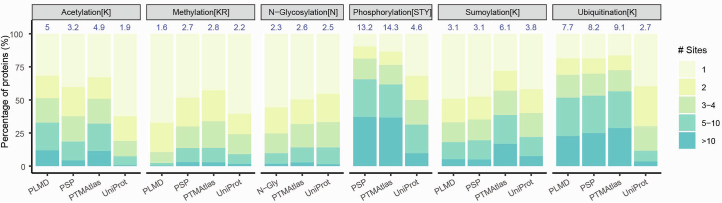
**Distribution of the number of PTM sites per protein across different PTMs in PTMAtlas compared to other databases**.

We compared PTMAtlas with several public databases, including PSP^13^, UniProt^19^, PLMD^20^ and N-GlycositeAtlas^21^, across the six PTM types. PTMAtlas contained the largest number of sites for acetylation, methylation, sumoylation and ubiquitination (Fig. 2e–j). For phosphorylation, it included five times as many sites as UniProt and added 83,162 sites that were not found in PSP (Fig. 2h). Notably, PSP applies less-stringent inclusion criteria, resulting in more phosphorylation sites but a potentially higher false positive rate^14^. Across all PTM types, PTMAtlas also had a higher average number of sites per protein than did other databases (Extended Data Fig. 2). These results underscore the comprehensive coverage and high quality of PTMAtlas.

### Deep-learning-based PTM site prediction

**Extended Data Fig. 3:**
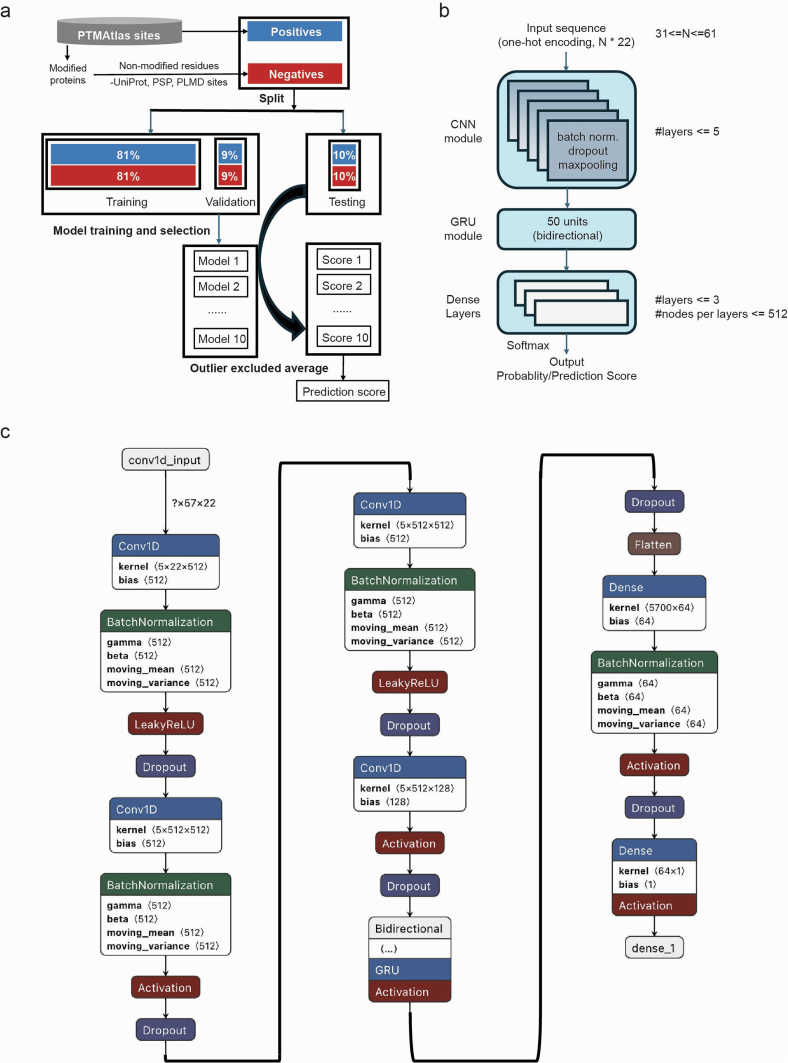
Deep learning framework for protein PTM site prediction. **a** Overview of the model training and testing workflow. **b** Model architecture design with the search space of key hyperparameters. **c** An example architecture of a phosphorylation model.

We used PTMAtlas to train DeepMVP, a set of enzyme-agnostic deep learning models, each specialized in site prediction for one of the six PTM types (Methods). For each type, positive sites were drawn from PTMAtlas; negatives were sampled from the same amino acid residues in PTMAtlas proteins, excluding the PTM sites listed in PTMAtlas, UniProt, PSP and PLMD. Ninety per cent of the data was used for training (81%) and validation (9%), and the remaining 10% was used for independent testing (Extended Data Fig. 3a). All reported results are based on the independent test set.

DeepMVP processes raw protein sequences by extracting a segment of length *N* (31–61 residues) centered on the target site, which serves as the sole input feature. The optimal *N* was treated as a hyperparameter and determined during training. We used a genetic algorithm to optimize neural architectures combining CNNs and bidirectional GRUs^22^ (Extended Data Fig. 3b,c). To ensure a robust final prediction, the top ten models based on validation accuracy were selected, and the final score was computed as the outlier-excluded average of these models (Extended Data Fig. 3a and Methods).

**Fig. 3:**
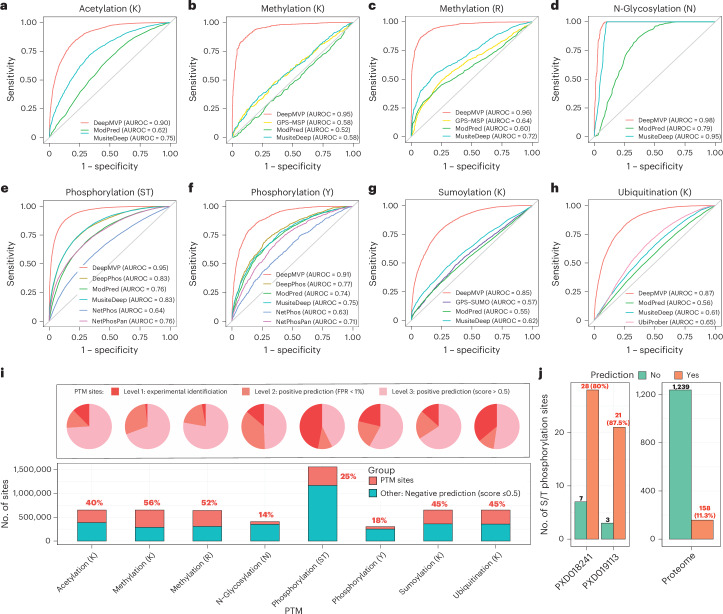
Evaluation and application of the DeepMVP for PTM site prediction. **a**–**h**, Performance comparison between DeepMVP and other models for site prediction for six PTM types, evaluated on the basis of AUROC. **i**, Proportion of PTM sites nominated in this study in the human proteome for the six PTM types and their classification into three confidence levels. **j**, Comparison between S/T phosphosite prediction results against phosphosites detected in two phosphoproteomic experiments, and the whole proteome of SARS-CoV-2. Source data

We benchmarked DeepMVP against eight established tools with publicly available pretrained models. MusiteDeep^11^ and ModPred^23^ support all six PTMs, whereas GPS-MSP^24^, DeepPhos^12^, NetPhos^25^, NetPhosPan^26^, GPS-SUMO^27^ and UbiProber^28^ each target a single PTM type. All models were evaluated on the 10% held-out independent test set. Across all six PTM types, DeepMVP outperformed all others in terms of the area under the receiver operating characteristic (AUROC) (Fig. 3a–h), achieving AUROC > 0.85 for every PTM and > 0.90 for acetylation, N-glycosylation, methylation and phosphorylation. Its exceptionally high AUROC for N-glycosylation (0.98) likely reflects the strong motif specificity.

**Extended Data Fig. 4:**
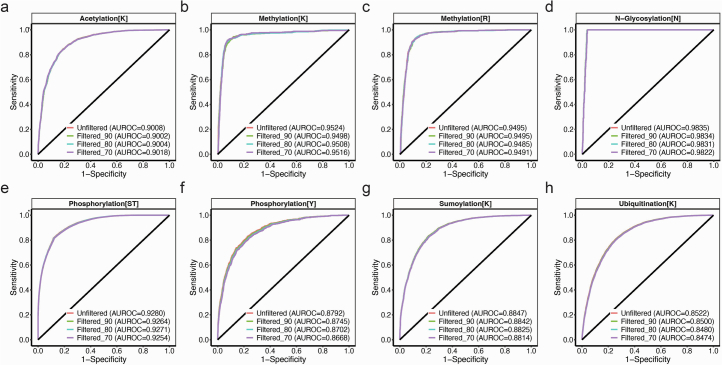
Evaluation of potential overfitting caused by sequence similarity between training and testing data. **a**–**h** PTM site prediction performance of DeepMVP for six PTM types using new training and testing datasets created with a protein-level split and controlled sequence similarity thresholds.

To assess potential overfitting from sequence similarity between training and test data, as has previously been observed in deep sequence-based prediction studies of protein–protein interactions^29^, we re-split the data at the protein level and excluded test peptides exceeding 90%, 80% or 70% sequence similarity to the training set in separate evaluations. AUROC values remained stable across all PTMs, with changes < 0.03 compared to the original models (Extended Data Fig. 4), confirming the robustness of DeepMVP under strict sequence-similarity control.

**Extended Data Fig. 5:**
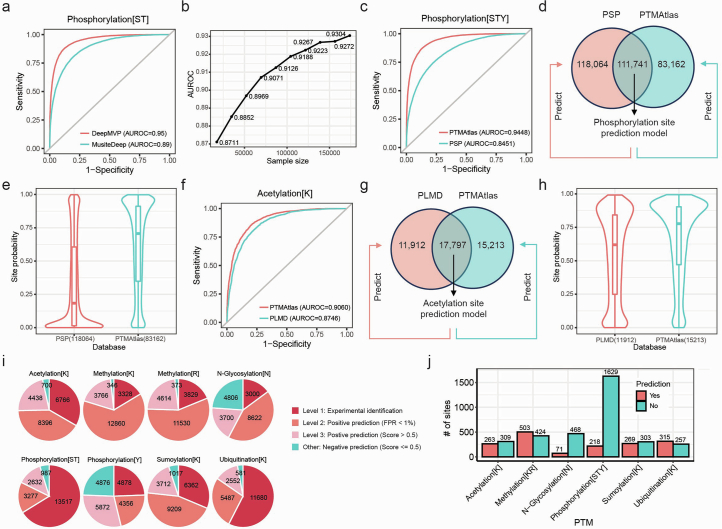
Factors impacting model performance and additional data on model application to the human and SARS-CoV-2 proteomes. **a** Comparative performance evaluation of MusiteDeep and our model for S/T phosphorylation prediction using identical training and testing data. **b** Analysis of the impact of increasing training data size on prediction performance for phosphorylation. **c** Performance comparison between models trained and tested with data from PTMAtlas or PSP for S/T/Y phosphorylation. **d**, **e** Application of phosphorylation site prediction models trained with the intersection data of PTMAtlas and PSP to the unique data from these two databases (**d**), along with the distribution of predicted probability scores (**e**). **f** Performance comparison between models trained and tested using data from PTMAtlas or PLMD for acetylation. **g**, **h** Application of acetylation site prediction models trained with the intersection data of PTMAtlas and PLMD to the unique data from these two databases (**g**), along with the distribution of predicted probability scores (**h**). **i** Proteome-wide classification of human proteins based on evidence of PTM sites at various confidence levels. **j** Numbers of predicted PTM sites in the SARS-CoV-2 proteome. Box and whisker plots present the means (lines inside the boxes), the 1st and 3rd quartiles (bottom and top bounds of the boxes), and the extents of the data (whiskers).

Among the published tools, MusiteDeep and DeepPhos, both based on deep learning, performed reasonably well but fell short of DeepMVP for all PTMs except N-glycosylation. To evaluate the contribution of model architecture versus training data, we retrained MusiteDeep for S/T phosphorylation using the same dataset as was used for DeepMVP. The retrained model achieved an AUROC of 0.89, still below DeepMVP’s 0.95 (Extended Data Fig. 5a), highlighting the advantage of our network design. Nevertheless, this retraining increased MusiteDeep’s AUROC from its original 0.83 (Fig. 3e), underscoring the critical role of high-quality training data in model performance.

To further elucidate how training data affect model performance, we conducted controlled analyses using various phosphorylation datasets while maintaining a consistent training procedure. First, we generated ten incrementally larger training sets from PTMAtlas by increasing data size in 10% steps, observing steady performance gains as the amount of training data increased (Extended Data Fig. 5b). We then compared phosphorylation models trained on PTMAtlas versus PSP. Despite PSP having more phosphosites (229,805) than PTMAtlas (194,903), the PSP-trained model achieved an AUROC of only 0.84 on its own test set, compared with 0.94 from the model trained and tested on PTMAtlas (Extended Data Fig. 5c). The lower performance of the PSP model likely reflects its lower data quality due to less-stringent site-inclusion criteria^14^. Supporting this, models trained on data shared between PTMAtlas and PSP achieved an average site probability above 0.7 for PTMAtlas-specific data, but below 0.25 for PSP-specific data (Extended Data Fig. 5d,e). A similar trend was observed when comparing PTMAtlas and PLMD for acetylation (Extended Data Fig. 5f–h). These results highlight that both the quantity and quality of PTMAtlas data contribute to the superior performance of DeepMVP.

Leveraging DeepMVP’s strong predictive performance, we conducted proteome-wide predictions for all six PTM types. We used a threshold of 0.5 on the prediction score (ranging from 0 to 1) to classify sites as positive, and applied a more stringent 1% false positive rate (FPR) cutoff to define high-confidence predictions (Methods). This analysis substantially expanded coverage across all PTM types (Fig. 3i). Notably, 56% of lysine and 52% of arginine residues were experimentally identified or computationally predicted as methylation sites. Coverage was slightly lower for acetylation (40%), sumoylation (45%) and ubiquitination (45%), followed by S/T phosphorylation (25%), tyrosine phosphorylation (18%) and N-glycosylation (14%). At the protein level, more than 99% of proteins were predicted to be modifiable (Extended Data Fig. 5i).

DeepMVP’s utility extends beyond human proteins to viral proteins, which are modified by host enzymes. When applied to the SARS-CoV-2 proteome, DeepMVP predicted 263 acetylation sites, 218 phosphorylation sites, 71 N-glycosylation sites, 503 methylation sites, 269 sumoylation sites and 315 ubiquitination sites (Extended Data Fig. 5j and Supplementary Table 3). All viral proteins were predicted to be modifiable. Among all ST residues in the SARS-CoV-2 proteome, 11.3% were predicted to be phosphorylation sites. Notably, DeepMVP recovered 80% and 87.5% of the experimentally identified S/T phosphorylation sites from two MS-based studies, representing 7.1- and 7.7-fold enrichments, respectively, over the baseline prediction rate of 11.3% (Fig. 3j). These results provide strong independent support for DeepMVP’s predictive accuracy.

Overall, DeepMVP outperformed existing PTM-site-prediction tools by leveraging both advanced neural network design and extensive, high-quality training data from PTMAtlas. This combination enabled comprehensive PTM site prediction across both human and viral proteomes.

### Predicting variant effects on PTMs

To evaluate DeepMVP’s performance in predicting variant effects on PTMs, we compiled 235 experimentally validated variant–PTM pairs through manual review of the literature, including only variants affecting PTM status within seven residues. These pairs involved 226 variants and 183 PTM sites across 126 proteins, comprising 119 phosphorylation, 55 sumoylation, 46 N-glycosylation, 12 methylation and 3 ubiquitination events (Supplementary Table 4). We grouped them according to variant proximity and effect direction, creating four categories: direct increase, direct decrease, proximal increase and proximal decrease.

**Fig. 4:**
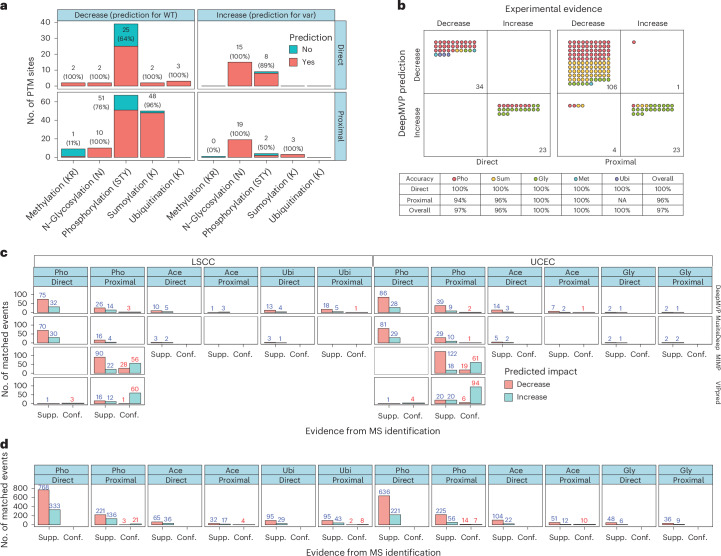
Validation of variant effect prediction using literature-curated PTM-altering variants and proteogenomic datasets. **a**, The sensitivity of DeepMVP in predicting PTM sites was assessed using the peptide with stronger experimental evidence for modification within a WT–variant (var) peptide pair, including WT peptide for variants known to decrease PTM (left) and var peptide for variants known to increase PTM (right). PTM sites were further categorized as overlapping with the variant position (direct, top) or located at a neighboring position relative to the variant (proximal, bottom). **b**, Directional concordance between effects predicted by DeepMVP and experimentally observed effects for the 191 variant–PTM pairs with correctly predicted PTM sites (from **a**). Pho, phosphorylation; Met, methylation; Sum, sumoylation; Ubi, ubiquitination (Ub); Gly, N-glycosylation; Ace, acetylation. **c**, Comparison of performance of DeepMVP and other tools on variant effect prediction, using two CPTAC cohorts (LSCC and UCEC). Predictions (increase or decrease) were separated into those supported (Supp., blue numbers) and conflicting (Conf., red numbers) on the basis of MS detection. This analysis was based on the first two batches of TMT experiments. **d**, Classification of DeepMVP’s predictions into the Supp. and Conf. groups on the basis of the complete CPTAC datasets. Source data

We first evaluated DeepMVP’s ability to predict PTM sites using the sequence with stronger experimental evidence (WT for decreased PTMs and variant for increased PTMs), as the alternative sequence might not correspond to a bona fide PTM site. A prediction was considered correct if the modifiable residue scored above 0.5. DeepMVP correctly predicted 191 of 235 events (81%) (Fig. 4a), showing comparable sensitivity for both direct and proximal variants.

We next evaluated DeepMVP’s accuracy in predicting the direction of variant effects using the 191 variant–PTM pairs with correctly predicted PTM sites. Delta scores below or above zero from the predictions were classified as ‘decrease’ or ‘increase,’ respectively (Fig. 1), and compared with experimentally observed effects. Accuracy was defined by directional consistency. As expected, DeepMVP achieved 100% accuracy for direct events. For proximal events, accuracy was also high, ranging from 94% to 100% across PTM types, resulting in an overall accuracy of 97% when combining both direct and proximal events (Fig. 4b).

To overcome biases in literature-curated PTM-altering variants, which often focus on well-studied variants and PTM sites, we used proteogenomic datasets from the Clinical Proteomic Tumor Analysis Consortium (CPTAC), providing both variant and PTM data from the same samples and enabling more comprehensive and unbiased evaluation. We analyzed two cancer cohorts—uterine corpus endometrial carcinoma (UCEC)^30^ and lung squamous cell carcinoma (LSCC)^31^—which include samples with a high mutation burden. Each cohort contains three MS/MS-based PTM datasets, and together they encompass four PTM types: phosphorylation, acetylation, N-glycosylation and ubiquitination. Sample-specific variants were used to construct customized protein databases, enabling PTM site identification on both WT and variant peptides (Methods). In parallel, DeepMVP was used to predict PTM effects for the same set of variants. We used a delta score cutoff of 0.5 to identify PTM-altering variants, which ensures that the predicted PTM probabilities for the WT and variant peptides were opposite sides of the 0.5 threshold used to classify sites as modifiable (positive) or not (negative). MS/MS detection of a PTM site exclusively on the variant peptide supported an increase prediction, whereas detection exclusively on the WT peptide supported a decrease prediction. Detections that contradicted the predicted direction were classified as conflicting. For benchmarking, we compared the results with those of MusiteDeep, and for phosphorylation predictions, we also included MIMP^9^ and VIPpred^32^ (Methods). Owing to the long runtime of some tools, comparisons were limited to the first two TMT (tandem mass tag) multiplexing batches per dataset.

Across both cohorts, DeepMVP identified more MS/MS-supported PTM-altering events than did MusiteDeep for most PTM types, except for N-glycosylation, for which both tools performed similarly (Fig. 4c). Both reported few conflicting events, indicating high specificity. MIMP and VIPpred, which are not designed to detect direct effects, identified more proximal phosphorylation-altering events but also produced significantly more conflicting calls, suggesting lower specificity (Fig. 4c).

After establishing DeepMVP’s balanced sensitivity and specificity, we applied it to all TMT batches across all six datasets, identifying 3,365 PTM-altering events, among which 3,296 (98%) were supported by MS/MS data and only 69 (2%) were conflicting (Fig. 4d). Together, these results underscore DeepMVP’s effectiveness and reliability in predicting variant effects on PTMs with strong agreement with experimental data.

### PTM effect prediction for pathogenic germline variants

**Fig. 5:**
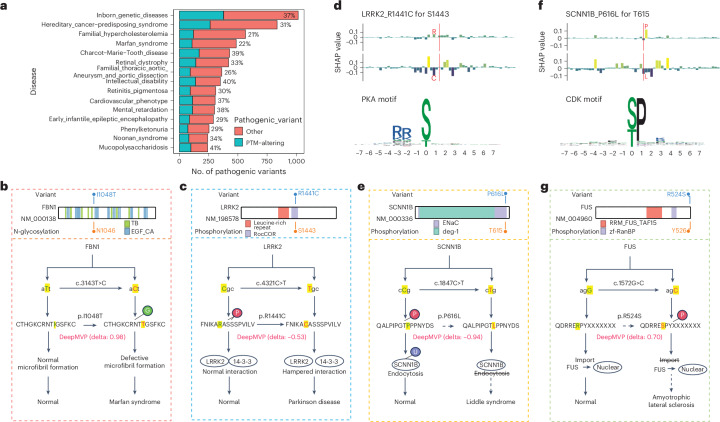
DeepMVP prediction of PTM-altering pathogenic germline variants. **a**, Ratio of PTM-altering variants predicted in the top 15 diseases with the highest number of associated variants annotated in ClinVar. **b**, Predicted impact and existing knowledge of the Marfan syndrome-associated substitution p.I1048T on *FBN1*. **c**, Predicted impact and existing knowledge of the Parkinson’s disease-associated substitution p.R1441C on *LRRK2*. **d**, Shapley value distribution of flanking amino acids for predicting phosphorylation site S1443 on LRRK2, with and without p.R1441C. **e**, Predicted impact and existing knowledge of the Liddle syndrome-associated substitution p.P616L on *SCNN1B*. **f**, Shapley value distribution of flanking amino acids for predicting phosphorylation site T615 on SCNN1B, with and without p.P616L. **g**, Predicted impact and existing knowledge of the amyotrophic lateral sclerosis-associated variant p.R524S on *FUS*. Source data

To uncover the functional links between genetic variants and disease phenotypes, we used DeepMVP to predict the impact of pathogenic germline variants on PTMs. For interpretable insights into these predictions, we used Shapley value analysis, based on cooperative game theory^33^, to quantify each amino acid’s contribution to the PTM prediction (Methods). We curated 24,237 pathogenic variants in canonical proteins from ClinVar for downstream analysis (Methods). Using a delta score threshold of 0.5, DeepMVP identified 7,713 variants (32%) that significantly impact 12,435 PTM events, including 4,602 proximal events (Supplementary Table 5). Among the 15 disease categories with the most pathogenic variants, the proportion of PTM-altering variants ranged from 21% to 41% (Fig. 5a). The highest counts were associated with ‘Inborn_genetic_diseases’ (378 variants), followed by ‘Hereditary_cancer-predisposing_syndrome’ (263), ‘Charcot–Marie–Tooth disease’ (169), ‘Retinal_dystrophy’ (141) and ‘Intellectual_disability’ (139).

**Extended Data Fig. 6:**
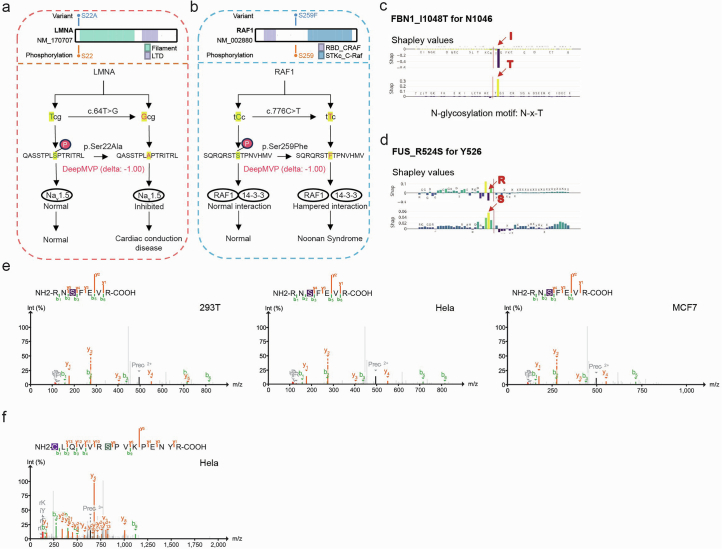
Additional data on DeepMVP prediction of PTM-altering pathogenic germline variants and somatic mutations. **a**, **b** Predicted impact and existing knowledge of the cardiac conduction disease-associated variant S22A on LMNA (**a**) and the Noonan syndrome-associated variant S259F on RAF1 (**b**). **c**, **d** Shapley value distribution of flanking amino acids for predicting glycosylation site N1046 on FBN1, with and without the I1048T variant (**c**) and for predicting phosphorylation site Y526 on FUS, with and without the R524S variant (**d**). **e** Example MS/MS spectra supporting the identification of TP53 p.S269 in 3 cell lines expressing TP53 G266R mutation. **f** An example MS/MS spectrum from supporting the identification of VHL p.S168 in Hela cell expressing VHL L169P mutation.

Some DeepMVP predictions—both direct and proximal—were supported by earlier experimental data and known regulatory mechanisms, underscoring the model’s accuracy. For example, DeepMVP confidently predicted phosphorylation at S22 of lamin A/C (LMNA), with the S22A variant yielding a delta score of –1.00 (Extended Data Fig. 6a). This agrees with studies showing that S22 phosphorylation regulates Na_v_1.5 function, and its loss contributes to cardiac conduction disease^34^. Similarly, DeepMVP predicted that phosphorylation at S259 on RAF1 would be abolished by the S259F variant (delta score, –1.00, Extended Data Fig. 6b); this aligns with earlier findings that S259 is an inhibitory site, and its dephosphorylation activates RAF1–ERK signaling, contributing to Noonan syndrome^35^.

As an example of proximal effect prediction, the Fibrillin-1 gene *FBN1*^I1048T^ was predicted to introduce a new N-glycosylation site at N1046 (delta score, 0.98). Shapley value analysis revealed the formation of a canonical NXS/T motif (Fig. 5b and Extended Data Fig. 6c). This variant is linked to Marfan syndrome and disrupts microfibril formation by introducing an N-glycosylation site at position 1046 (ref. ^36^). In another example, *LRRK2*^R1441C^, which is associated with Parkinson’s disease, was predicted to have reduced phosphorylation at S1443 (delta score, –0.53, Fig. 5c); Shapley value analysis suggested that a PKA-recognition motif was disrupted (Fig. 5d). This matches experimental evidence showing that p.R1441C impairs PKA-mediated phosphorylation of S1443, disrupts LRRK2’s interaction with 14-3-3 proteins and modulates its kinase activity^37^ (Fig. 5c), reinforcing a known pathogenic mechanism in Parkinson’s disease.

In contrast to the examples above, many PTM-altering pathogenic germline variants predicted by DeepMVP have not yet been reported. For instance, DeepMVP predicted that the p.P616L variant in *SCNN1B*, which encodes the β-subunit of the epithelial sodium channel (ENaC), would be associated with reduced phosphorylation at T615 (delta score, –0.94; Fig. 5e). Although direct experimental validation is lacking, this prediction is supported by known biology: P616L is linked to hypertension and Liddle syndrome through ENaC overactivation^38^, whereas T615 phosphorylation is known to trigger ubiquitination and internalization of ENaC^39^. Loss of phosphorylation at this site could therefore prolong ENaC surface expression and contribute to disease. Shapley value analysis suggested the loss of a canonical S/TP motif recognized by CDK kinases (Fig. 5f), providing regulatory insight that can be experimentally validated.

In another instance, DeepMVP predicted that the R524S variant in the RNA-binding protein FUS, a variant associated with amyotrophic lateral sclerosis (ALS), would be associated with enhanced phosphorylation at Y526 (delta score, 0.70; Fig. 5g). This variant disrupts FUS binding to its nuclear import receptor, resulting in cytoplasmic mislocalization^40^. Phosphorylation at Y526 by Src family kinases also impairs nuclear import^41^, supporting the hypothesis that p.R524S promotes Y526 phosphorylation and contributes to ALS pathology. Moreover, Src inhibition could be investigated as a viable therapeutic approach for people with ALS carrying FUS-R524S. Although Y526 is located at the carboxy terminus of the protein and does not conform to the conventional YXXL/I/V motif typically recognized by Src family kinases, our analysis of Shapley value distribution highlighted the significance of the preceding ESP amino acid sequence for this phosphorylation event (Extended Data Fig. 6d).

In summary, DeepMVP identified thousands of high-confidence PTM-altering pathogenic germline variants. Although some predictions were supported by earlier studies, others offer new functional hypotheses linking uncharacterized variants to disease through PTM dysregulation. Moreover, the application of Shapley value analysis not only made our predictions interpretable, but also facilitated connection of altered PTMs to possible modifying enzymes that could be therapeutically targetable.

### Pan-cancer analysis of somatic mutations’ impact on PTMs

**Fig. 6:**
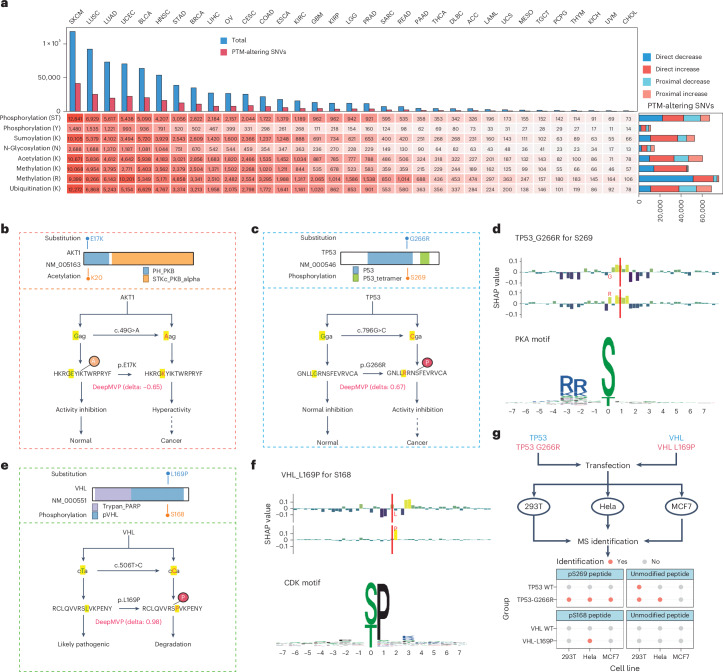
DeepMVP prediction of PTM-altering somatic mutations across 33 cancer types. **a**, Distribution of the predicted PTM-altering somatic mutations across 33 cancer types. SKCM, skin cutaneous melanoma; LUSC, lung squamous cell carcinoma; LUAD, lung adenocarcinoma; UCEC, uterine corpus endometrial carcinoma; BLCA, bladder urothelial carcinoma; HNSC, head and neck squamous cell carcinoma; STAD, stomach adenocarcinoma; BRCA, breast invasive carcinoma; LIHC, liver hepatocellular carcinoma; OV, ovarian serous cystadenocarcinoma; CESC, cervical squamous cell carcinoma and endocervical adenocarcinoma; COAD, colon adenocarcinoma; ESCA, esophageal carcinoma; KIRC, kidney renal clear cell carcinoma; GBM, glioblastoma multiforme; KIRP, kidney renal papillary cell carcinoma; LGG, brain lower grade glioma; PRAD, prostate adenocarcinoma; SARC, sarcoma; READ, rectum adenocarcinoma; PAAD, pancreatic adenocarcinoma; THCA, htyroid carcinoma; DLBC, lymphoid neoplasm diffuse large B-cell lymphoma; ACC, adrenocortical carcinoma; LAML, acute myeloid leukemia; UCS, uterine carcinosarcoma; MESO, mesothelioma; TGCT, testicular germ cell tumors; PCPG, pheochromocytoma and paraganglioma; THYM, thymoma; KICH, kidney chromophobe; UVM, uveal melanoma; CHOL, cholangiocarcinoma. SNV, single-nucleotide variant. **b**,**c**, Predicted impact and existing knowledge of the substitutions p.E17K on *AKT1* (**b**) and p.G266R on *TP53* (**c**). **d**, Shapley value (SHAP) distribution of flanking amino acids for predicting phosphorylation site S269 on p53, with and without p.G266R. **e**, Predicted impact and existing knowledge of the substitution p.L169P on *VHL*. **f**, Shapley value distribution of flanking amino acids for predicting S168 phosphorylation on VHL, with and without p.L169P. **g**, Identification of pS269 in p53 and the corresponding unmodified peptide in cell lines expressing TP53 WT or TP53-G266R, as well as pS168 in VHL and the corresponding unmodified peptide in cell lines expression VHL WT or VHL-L169P. Source data

To investigate the functional impact of somatic mutations in cancer, we used DeepMVP to analyze 791,637 somatic missense mutations from 9,079 samples spanning 33 cancer types^16^. Using a delta score threshold of 0.5, 230,092 mutations (31%) were predicted to alter PTMs, with rates ranging from 27% to 35% across cancer types (Fig. 6a). R-methylation had the highest number of PTM-altering mutations, whereas N-glycosylation and Y-phosphorylation had the fewest, likely reflecting fewer confidently predicted sites (Fig. 3i). Most predicted mutation–PTM pairs were direct, particularly for methylation (Fig. 6a). Notably, mutations were more often predicted to increase, rather than decrease, PTM levels, except for S/T phosphorylation and R-methylation.

Similar to our analysis of germline variants, DeepMVP revealed new functional insights into somatic mutations. For instance, in AKT1, the E17K hotspot substitution, known to drive sustained activation of the protein in multiple cancers^42^, was predicted to reduce acetylation at K20 (delta score, –0.65; Fig. 6b). Acetylation at K20, detected by MS in HeLa cells, has been shown to inhibit AKT1 activity^43^. Moreover, p.E17K failed to promote AKT1 membrane localization or activation when p.K20Q was present to mimic the effects of acetylation^43^. These findings support a mechanistic link in which p.E17K activates AKT1 by reducing K20 acetylation, as predicted by DeepMVP.

In another example, DeepMVP predicted that p.G266R in TP53 would increase phosphorylation at S269 (delta score, 0.67; Fig. 6c). Shapley value analysis suggested that this increase was driven by the formation of an RRXS/T motif, which is recognized by PKA kinases (Fig. 6d). Although p.G266R is a known loss-of-function substitution in TP53 (refs. ^44,45^), and S269 phosphorylation has been reported to inhibit TP53 activity^46^, a direct connection between this substitution and the PTM had not been established. DeepMVP bridges this gap by offering a functional hypothesis linking p.G266R to S269 phosphorylation and identifying a potential upstream kinase.

As a third example, DeepMVP predicted that p.L169P in the tumor-suppressor VHL, recurrent in clear cell renal carcinoma but with unclear functional consequences^47^, would increase phosphorylation at S168 (delta score, 0.98; Fig. 6e). Shapley value analysis indicated that this was likely owing to the creation of an S/TP motif, recognized by proline-directed kinases such as cyclin-dependent kinases (CDKs) (Fig. 6f). This phosphorylation is known to promote ubiquitination and degradation of VHL^48^; DeepMVP thus connects p.L169P to altered phosphorylation and potential upstream kinase regulation.

**Extended Data Fig. 7:**
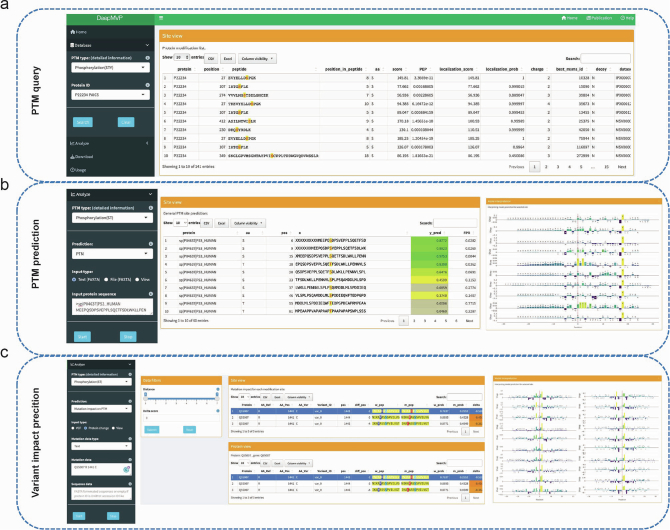
Interface and functionalities of the DeepMVP web server. **a** PTMAtlas query interface. **b** PTM site prediction interface. **c** Variant impact prediction interface.

To experimentally validate DeepMVP predictions, WT and mutated forms of *TP53* and *VHL* were introduced through lentiviral expression vectors into three commonly used cell lines: MCF7, 293T and HeLa. Phosphorylated peptides were enriched and analyzed by liquid chromatography–MS/MS. Although p53 peptides with unmodified S269 were detected in one *TP53* WT cell line and two *TP53*^G266R^ cell lines, p53 peptides with phosphorylated S269 were observed in all three cell lines expressing *TP53*^G266R^ but not in WT control cells (Fig. 6g and Extended Data Fig. 6e). For VHL, phosphorylation at S168 was identified exclusively in Hela cells expressing the L169P variant, with no unmodified peptides observed in any samples (Fig. 6g and Extended Data Fig. 6f). These experimental data were consistent with DeepMVP’s predictions. To support broader adoption and validation, we have made DeepMVP available through a user-friendly web-server (Extended Data Fig. 7) and a Python package, enabling researchers to explore PTM effects of genetic variants.

## Discussion

Missense variants can contribute to disease by altering PTMs, key regulators of protein function. However, knowledge of PTM-modifying variants remains limited, with existing computational predictions largely confined to phosphorylation mediated by well-characterized kinases. DeepMVP enables proteome-wide prediction of variant effects across six major PTM types, identifying 7,713 PTM-altering pathogenic germline variants and 230,092 somatic mutations in cancer. These predictions enable assessment of the functional consequences of missense variants through the lens of PTMs, offering a potential functional link between genotype and disease phenotype. Although DeepMVP is enzyme-agnostic, its interpretability allows inference of potential modifying enzymes, with implications for development of therapeutics.

DeepMVP substantially outperforms previously published models for enzyme-agnostic PTM site prediction, including both traditional machine learning and deep learning approaches—the latter generally performing better than the former. Its improved performance over other deep learning models can be partially attributed to the integration of CNN and GRU architectures, the use of a genetic algorithm to optimize network design and model ensembling to enhance robustness. CNNs capture local sequence patterns, whereas GRUs model residue-level dependencies, together enabling accurate prediction of PTM site determinants. Although we explored transformer-based architectures early in development, they did not exceed the performance of the CNN/GRU model and required substantially more computational resources.

DeepMVP also benefits considerably from its high-quality training dataset derived from PTMAtlas, which represents the most comprehensive and rigorously curated collection of PTM sites to date. We demonstrate that both the quantity and quality of training data have a substantial impact on model accuracy. In addition to predicting PTM sites, PTMAtlas is a valuable resource for the broader research community, with the potential to support diverse investigations such as those exploring PTM cross-talk, hotspot identification and PTM–domain or PTM–structure interactions. Although these applications are beyond the scope of this study, PTMAtlas is publicly available to facilitate such efforts.

One limitation of this study arises from the ‘tryptic bias’ in the positive training data, a common issue for all models trained on MS/MS data. Most MS/MS workflows use trypsin to digest proteins into optimal-length peptides for detection, but PTM sites on tryptic peptides that are too short or too long often go undetected. This limitation contributes to training bias and false negative predictions, such as the missed phosphorylation site S106 on ESR1, which resides on a 96-amino-acid tryptic peptide. The community could generate deep PTM-enriched datasets using alternative proteases to mitigate this effect.

Our modeling approach has additional limitations, including restricted sequence length and simplified sequence representation. Although PTM enzyme specificity is largely determined by local sequence context, broader protein context can influence modification potential. For example, PTMs are unlikely to occur at structurally buried sites. Without incorporating such contextual information, our models might produce false positives. Transformer-based protein language models, such as ESM-2 (ref. ^49^), generate embeddings that implicitly capture evolutionary, functional and structural features from sequence data. These can be complemented by explicit three-dimensional structural information from tools such as AlphaFold^50^. Integrating these complementary sources into a unified framework could substantially enhance the accuracy and contextual awareness of PTM site prediction.

Despite these limitations, our systematic evaluation using literature-curated PTM-altering variants and proteogenomics datasets demonstrated strong concordance between DeepMVP predictions and experimental data, underscoring its reliability. Although many computational tools distinguish pathogenic from benign variants^51–54^, they often lack the resolution to predict specific functional consequences. DeepMVP fills this gap by identifying PTM alterations as direct outcomes of missense variants, providing a crucial functional bridge between genotype and clinical phenotype. By revealing how variants drive disease through PTM dysregulation, DeepMVP offers a promising framework for advancing our understanding of variant function in human biology and disease.

## Methods

### Human PTM-enriched MS/MS datasets

A total of 241 human PTM-enriched MS/MS datasets were downloaded from PRIDE (https://www.ebi.ac.uk/pride/), PDC (https://proteomic.datacommons.cancer.gov/pdc/), iProX (http://www.iprox.org), MassIVE(https://massive.ucsd.edu), Chorus (https://chorusproject.org) and PeptideAtlas (https://peptideatlas.org), including 157 datasets for phosphorylation, 18 for acetylation, 16 for ubiquitination, 14 for sumoylation, 20 for methylation and 16 for glycosylation (Supplementary Table 1). The glycosylation data were obtained from experiments involving glycan removal, resulting in datasets that lack glycan structure information and focus exclusively on the presence or absence of glycosylation at specific sites. Among the 157 phosphoproteomics datasets, 110 were sourced from a meta dataset (PXD012174), in which all raw data were downloaded from PRIDE and jointly analyzed using MaxQuant^17^. For these datasets, we downloaded the MaxQuant search result from PRIDE under the accession number PXD012174. For all other datasets, the raw MS/MS data were downloaded and analyzed in this study.

### MS/MS data analysis and quality control

All downloaded raw MS/MS data were reanalyzed using MaxQuant^17^ (v1.6.5.0). The MS/MS data were searched against reviewed human protein sequences from UniProt (downloaded 14 February 2019, 20,413 sequences in total), and common laboratory-contaminant proteins were added using MaxQuant. The common contaminant proteins were provided by MaxQuant. Each dataset was separately analyzed using MaxQuant. For each dataset, the search parameters, including enzyme type and fixed and variable modifications, were determined on the basis of the experiment protocol used to generate the data. For all searches, the minimum peptide length was set to seven amino acids. For the identification of modified peptides, the default MaxQuant search parameters were used, including 1% FDR at PSM, protein and site levels, a minimum Andromeda score of 40 and a minimum delta score (the score difference between the best and second best matching candidate for an MS/MS spectrum) of 6. FDRs were estimated using a target–decoy strategy^18^. For each dataset, the site table generated by MaxQuant (for example, Phospho (STY)Sites.txt for phosphorylation) was used for downstream analysis. Complete search parameters for all datasets are available as xml files in the download section at http://deepmvp.ptmax.org.

For each type of PTM, all results from the site table files were combined, and a global site-level FDR across all the datasets was calculated using PGA^55^. A threshold of 1% global site-level FDR was applied, along with an additional filter requiring a site localization probability greater than 0.5. For N-linked glycosylation, a filter by the N!PS/T motif was also applied. All sites that passed the filtering steps described above were included in the database PTMAtlas and used for downstream analysis. They can be accessed in the download section at http://deepmvp.ptmax.org.

### Public PTM site databases

PTM sites from four public databases were used for comparison. For phosphorylation, sites with modification occurring on amino acid S, T or Y from PhosphoSitePlus^13^ (downloaded 4 March 2020) and UniProt (downloaded 14 February 2019) were used. For methylation, sites with modification occurring on amino acid K or R from PhosphoSitePlus, UniProt and PLMD^20^ (downloaded 4 March 2020, v3.0) were used. For acetylation, sumoylation and ubiquitination, sites with modification occurring on amino acid K from PhosphoSitePlus, UniProt and PLMD were used. For N-linked glycosylation, sites with modification occurring on amino acid N from N-GlycositeAtlas^21^ (downloaded 4 March 2020) and UniProt were used. For UniProt, only the sites annotated with the Evidence and Conclusion Ontology (ECO) ECO:0000269, ECO:0000305 or ECO:0000244 were used. For all databases, only sites from human proteins were used. All the sites from PhosphoSitePlus, PLMD and N-GlycositeAtlas were mapped to the same version of UniProt human protein database (downloaded 14 February 2019, 20,413 sequences in total), and only the mappable sites were used in this study.

### Deep-learning-based PTM site prediction

#### Training, validation, and testing data

Training, validation and testing data for enzyme-agnostic prediction of PTM sites contained positive samples and negative samples. Each sample is a peptide sequence, with the target PTM site positioned at the center and flanking sequences of equal length on both sides. For phosphorylation, the prediction sites were S, T and Y. For methylation, the prediction sites were K and R. For N-glycosylation, the prediction site was N. For acetylation, ubiquitination and sumoylation, the prediction site was K. For each type of PTM, the positive samples were defined as sites with MS/MS evidence in PTMAtlas; the negative samples were defined as sites of the same type that lacked MS/MS evidence for known PTMs from the same proteins. Only proteins with at least ten PTM sites were selected to generate negative samples, and samples already listed in the positive dataset or in existing databases, such as PSP or UniProt, were further removed from the negative dataset. For each type of PTM, all peptide sequences were randomly split into three distinct sets: 81% for training, 9% for validation during training and 10% for independent testing (Extended Data Fig. 3a). The training set was used to optimize the model, and the validation set helped tune hyperparameters and monitor performance on unseen data during training. The independent test set, untouched during training and validation, was used to evaluate the model’s final performance. The reported results are based on this independent test set.

To assess the potential for overfitting driven by sequence similarity between training and testing data (for example, due to peptides from the same protein or homologous sequences), we generated additional datasets for analysis. The modified proteins were initially split into three subsets, comprising 81%, 9% and 10% of the total, respectively, for the creation of training, validation and testing datasets. From each subset, 61-residue sequences centered on the modifiable sites, including both positive and negative sites, were extracted. To control for sequence similarity, peptides in the testing set were filtered to remove those with identities above predefined thresholds (90%, 80% or 70%) compared with peptides in the training and validation sets. This filtering was performed using the clustering tool CD-HIT (v4.8.1)^56^. The resulting filtered datasets, along with the original unfiltered dataset, were used to further evaluate model performance.

#### Deep learning framework

The deep learning framework for prediction of PTM sites in proteins is depicted in Extended Data Fig. 3. In brief, we treated the PTM site prediction problem as a binary classification problem, and for each type of PTM, ten deep-learning-based prediction models were developed using peptide sequences as the sole input feature. For model testing and application, scores from the ten models were combined using an outlier-excluded average, generating the final output confidence score of the PTM prediction (Extended Data Fig. 3a). Further details are provided below.

The framework takes raw protein sequences as input, and a sequence of length *N* (31 ≤ *N* ≤ 61), centered on the target site. The segment was extracted and encoded using one-hot encoding^57^, which includes 23 possible characters, representing the 20 standard amino acids, two uncommon amino acids (selenocysteine (U) and pyrrolysine (O)) and a padding character (X). If the flanking sequence was shorter than the specified *N*, it was padded with the X, which was represented as a vector of all zeroes, to ensure a consistent length. This process resulted in each PTM site being represented by an *N* × 22 matrix. Although most tools focus on shorter core motifs of approximately 15 amino acids, a longer sequence can capture additional structural and functional context surrounding the core motif, which can enhance prediction accuracy. To balance the additional contextual information gained from longer sequences with computational efficiency, we used sequences of up to 61 amino acids, centered on the PTM site. *N* was a hyperparameter that was optimized for each model during the training process described below.

The neural network architectures for each PTM were automatically designed using a genetic algorithm similar to the one used in our previous study^22^. Specifically, the genetic algorithm was designed to search different CNNs^58^ combined with GRU networks^59^, along with peptide sequence length for classification (Extended Data Fig. 3b). CNNs can capture local patterns in protein sequence data, whereas GRU networks can capture long-term dependencies between amino acids. The genetic algorithm initially created the first generation of individuals represented as deep neural networks and subsequently conducted genetic operations to facilitate their evolution in a genetic process. The genetic operations included selection, mutation and cross-over. Each neural network architecture was represented as a fixed-width genome encoding information about the network’s structure. In our setup, a model included a number of convolutional layers, a number of dense layers, an optimizer and a fixed bidirectional GRU layer with 50 units. The convolutional layers could be evolved to include varying numbers of feature maps, different activation functions, different kernel sizes, varying dropout proportions and whether to perform batch normalization and/or max pooling. Similar options were available for the dense layers, except for max pooling and kernel size. Additionally, the length of the flanking sequence of the target site was considered as a variable. The detailed search space is described in Supplementary Table 6. The quality of each network was assessed on the basis of its prediction accuracy on a validation dataset. Throughout the genetic process, we evaluated each individual network structure by training it from scratch. The process concluded after a fixed number of generations. By default, the generation size was 20, the population size for each generation was 50 and a maximum of 20 epochs and early stop were used for training.

During the neural network architecture search, only training and validation data were used. For each type of PTM, the top ten best neural architectures were selected on the basis of validation accuracy. Extended Data Fig. 3c illustrates the architecture chosen for one of the phosphosite prediction models. After the neural network architecture search, ten models were trained on the basis of the top ten best neural architectures from scratch, respectively, with a maximum of 100 epochs and a batch size of 64. The default learning rate was used for each model, and early stop was used in the training. The best-trained model, determined on the basis of validation accuracy, was saved for each architecture. The top ten models were ensembled using averaging with removal of outliers, determined using the interquartile range (IQR) algorithm. Specifically, the first quantile (Q1), the third quantile (Q3) and the interquartile range (IQR, that is, Q3–Q1) of the probabilities for each site were calculated, and probabilities outside of the boundaries of Q1 – 1.5 × IQR and Q3 + 1.5 × IQR were excluded. Then, the average was calculated from the remaining probabilities to represent the final probability for each site. The deep learning models were implemented using Python with Tensorflow (v2.4.0, www.tensorflow.org). The trained models for each type of PTM are available at https://github.com/bzhanglab/DeepMVP/.

#### Benchmarking of PTM site prediction

To evaluate the performance of DeepMVP models for PTM site prediction, we compared them with eight published tools for enzyme-agnostic PTM site prediction, including ModPred^23^, MusiteDeep^11^, GPS-MSP^24^, GPS-SUMO^27^, DeepPhos^12^, NetPhos (v3.1), NetPhosPan^26^ and UbiProber^28^. Among them, MusiteDeep and DeepPhos were also deep-learning-based tools. For all public tools, we used the pretrained models made available by the original developers in the comparison. For each type of PTM, the performance of all tested tools was evaluated using the independent testing data and quantified using AUROC.

For MusiteDeep, we also trained models using the same training data as DeepMVP and compared the performance of the two models using the same testing data.

### Proteome-wide predictions for human and SARS-CoV-2 proteomes

The FASTA file containing human protein sequences was downloaded from UniPort (accessed on 14 February 2019) and used as input to our deep learning models for proteome-wide PTM site prediction. Likewise, the FASTA file containing 28 SARS-CoV-2 protein sequences was downloaded from UniProt and used as input to DeepMVP for PTM site prediction. A probability score cutoff of 0.5 was applied to identify positive predictions across the six PTM types. Among these positive predictions, we further identified those with a probability score exceeding the threshold corresponding to a 1% false positive rate (FPR), as determined from the prediction results on the test data for each respective PTM type.

### SARS-CoV-2 phosphoproteomics data analysis

The two SARS-CoV-2 phosphoproteomics datasets were downloaded from PRIDE under accession numbers PXD019113 and PXD018241. The MS/MS data were searched using MaxQuant (version v1.6.5.0) against a protein database containing proteins from both *Chlorocebus aethiops* and SARS-CoV-2, with the following parameters: fixed modification, carbamidomethyl (C); variable modifications, oxidation (M), acetyl (protein amino terminus) and phospho (STY); the default enzyme ‘Trypsin/P’ was used, with a maximum of two missed cleavage sites. The protein sequences of *C. aethiops* were downloaded from UniProt. All other parameters were set to their default values. Only PTM sites with a localization probability above 0.75 were used for downstream analysis.

### Predicting variant effects on PTMs using DeepMVP

For each missense variant, we first identified potential PTM sites within the adjacent seven amino acids of the variant. These sites included S, T and Y for phosphorylation, N for N-glycosylation, K and R for methylation and K for acetylation, ubiquitination and sumoylation. Then, we used DeepMVP to predict the PTM probabilities for both the reference and variant sequences. To quantify the impact of the variant, we calculated a score as the absolute difference between these probability values:

$${P}_{{\rm{delta}}}={|P}({\rm{variant}})-P({\rm{reference}})|$$

*P*(variant) represented the probability of PTM occurrence at the specific site in the variant sequence, and *P*(reference) indicated the probability at the specific site in the reference sequence. If a variant occurred at a potential PTM site, we assigned *P*(variant) a value of zero when the potential PTM site was removed (for example, for phosphorylation, by changing S, T or Y to a different amino acid); *P*(reference) was set to zero when the variant led to the creation of a new potential PTM site (for example, for phosphorylation by changing a non-STY amino acid to one of those residues).

### DeepMVP evaluation using known PTM-altering variants

We performed an extensive manual literature review to compile a dataset of variants experimentally validated to impact PTMs. Given that most studies examine a limited range between the variant and the PTM site, this dataset includes only variants affecting PTM status within seven residues of the variant. We also attempted to construct a negative dataset of variants that do not affect neighboring PTMs. However, this proved challenging because negative results are rarely reported, and when they are, the experiments are always enzyme- and context-specific, and thus are not appropriate for evaluating our predictions, which are enzyme- and context-agnostic. It is nearly impossible to identify publications reporting a variant that does not affect a neighboring PTM site for modification by any enzyme of the class (for example, kinases) under any conditions.

To address this challenge, we adopted a two-step strategy. First, we used this dataset to evaluate the efficacy of DeepMVP in predicting PTM sites. For variants that decrease PTM, a probability score above 0.5 for the WT peptide sequence was considered a correct prediction. This threshold was applied to variant peptide sequences for variants that induce an increase. Next, for the variant–PTM pairs with correctly predicted PTM sites, we evaluated the accuracy of DeepMVP in variant effect prediction. This is possible because both the experimental data and our predictions are directional. The predicted effects were classified as decrease for delta scores below zero and increase for scores above zero. We then evaluated the performance of DeepMVP on the basis of the concordance between its prediction and experimental evidence.

### DeepMVP evaluation using proteogenomics data

For this analysis, we selected recently published proteogenomics data from two CPTAC cohorts that were not included in PTMAtlas. These cohorts were chosen for their inclusion of high-mutation-burden samples and diverse PTM data. The UCEC cohort^30^ includes phosphoproteome, acetylome and glycoproteome data, whereas the LSCC cohort^31^ contains phosphoproteome, acetylome and ubiquitylome data.

Somatic mutations were downloaded from the LinkedOmics database^60^ (https://www.linkedomics.org). Germline variants were identified using HaplotypeCaller from the Sarek pipeline^61^ and annotated with Ensembl Variant Effect Predictor (v110.0)^62^, using the same gene annotation as for somatic mutations. For each variant, 15-base peptides flanking the variant site were extracted from both reference and variant proteins to use as the input for DeepMVP and other tools, including MusiteDeep and two tools that predict variant effects on phosphorylation, MIMP^9^ and VIPpred^32^. We attempted to run other published tools for predicting variant effects on phosphorylation, but failed. Owing to the long runtimes of some tools, the comparative analyses were limited to the first two TMT batches, whereas DeepMVP analysis was performed for samples from all TMT batches. For DeepMVP and MusiteDeep, a delta score cutoff of 0.5 was used to determine PTM-altering variants. This cutoff was chosen because it provides a clear distinction and ensures that reference and variant peptides fall into different PTM site prediction categories (0–0.5, non-PTM site; 0.5–1, PTM site). For MIMP, a probability score of 0.5 was used, along with a requirement for a twofold difference between WT and mutant scores. When multiple predictions were available for a variant from different kinase models, the result with the highest probability score was selected. For VIPpred, default parameters were applied, and variants were classified into three categories: pairGain, pairLoss and pairNoimpact.

In parallel, raw MS/MS files for the six datasets from the two cohorts were downloaded from the Proteomic Data Commons (PDC, https://pdc.cancer.gov/pdc/). Customized reference databases were created for each TMT batch, incorporating variant proteins derived from somatic and germline variants in the corresponding samples and a common contaminant database from FragPipe. FragPipe V22.0 (ref. ^63^) pipelines from relevant proteomics platforms were then used to search protein databases, using customized databases for identifying PTM sites from both reference and variant proteins. For proteomics dataset searches, tryptic peptide lengths were set between 7 and 50 amino acids, allowing for up to 2 missed cleavages, with a precursor ion tolerance of 10 ppm and a fragment ion tolerance of 20 ppm. Carbamidomethylation (+57.0215 Da) on Cys and TMT modifications (+229.1629 Da) on the peptide N terminus and Lys were specified as fixed modifications. Oxidation (+15.9949 Da) on Met was included as a dynamic modification. Peptide identification was performed with a maximum FDR of 1% at the peptide level. For phosphoproteomics data, dynamic phosphorylation (+79.9663 Da) on Ser, Thr and Tyr was specified. For acetylproteomics data, dynamic acetylation (+42.0105 Da) and carbamylation (+43.0058 Da) on Lys were included. For ubiquitination proteomics data, ubiquitination was specified using two variable modifications on Lys: +114 (ubiqutination in addition to TMT on the same residue) and −115 (just ubiquitination, without TMT on the same residue). In addition, up to three missed cleavages and a maximum of four variable modifications per peptide were allowed. For glycoproteomics data searching, the Glycans-Medium-253 modification database was used. Identified PTM peptides covering a variant position were classified into three categories:

1. WT-only: PTM site identified exclusively on WT peptide.
2. MT-only: PTM site identified exclusively on variant peptide.
3. Both: PTM site identified on both WT and variant peptides.

Concordance between computational predictions and experimental data was evaluated by assessing whether variants that were predicted to have an increased impact on PTMs corresponded to a higher likelihood of detecting the associated PTM site on mutant peptides than on WT peptides, and vice versa for variants predicted to have a decrease impact. Because TMT quantifications are not directly comparable between different peptides, PTM sites identified on both WT and variant peptides (category 3) could not be reliably included in the evaluation. Moreover, all computational tools produced uncertain or no-impact predictions for some variants, complicating the use of standard performance evaluation metrics such as recall, precision and accuracy. Therefore, we relied on MT-only MS/MS detection as supporting evidence for increase predictions, and WT-only detection as supporting evidence for decrease predictions; the opposite detections were treated as conflicting evidence. To compare performance, we assessed both the number of predictions supported by MS/MS evidence, for which a higher count indicates higher sensitivity, and the number of predictions conflicting with MS/MS evidence, for which a lower count reflects higher specificity.

### Analysis of pathogenic variants and somatic mutations

Pathogenic variants were obtained from ClinVar (https://www.ncbi.nlm.nih.gov/clinvar/, downloaded 28 January 2021). The Ensembl Variant Effect Predictor^62^ (v102.0, https://useast.ensembl.org/info/docs/tools/vep/index.html) was utilized for variant annotation. When variants could be mapped to multiple transcripts of the same gene, only the variant mapped to the canonical protein was retained, to eliminate redundancy. Somatic mutations were downloaded from the TCGA data portals (https://portal.gdc.cancer.gov/, accessed 12 January 2020). The ANNOVAR (v20191024)^64^ tool was applied to annotate the functional consequence of all somatic mutations. Only missense variants were used for downstream analysis. For each variant, 15-base peptides flanking the variant site were extracted from both reference and variant proteins as the input of DeepMVP. A delta score cutoff of 0.5 was used to determine PTM-altering variants.

### Shapley value analysis

The Shapley value was computed using the function DeepExplainer from the Python package shap (https://github.com/shap/shap, version 0.39.0).

### Experimental evaluation of variant effect predictions

#### Plasmids

The WT and mutated target genes, including *TP53* and *VHL*, were cloned into Fucgw vector^65^ by Genscript Company. Plasmid DNAs were isolated with QIAfliter Plasmid Midi Kit (Qiagen,12243). The sequences were confirmed by Sanger sequencing (GENEWIZ).

#### Cell lines

MCF7, 293T and Hela cell lines were purchased from ATCC. All cell lines were cultured in DMEM (Corning,10-014-CM), with 10% FBS (Gibco, A5209401) and 1% penicillin–streptomycin (VWR,12001-692) in tissue culture incubators supplemented with 5% CO_2_.

#### Lentivirus package and cell infection

All lentiviruses were packaged in the 293T cell line using the jetPRIME transfection reagent (Polyplus,101000046), following the manufacturer’s instructions. The culture supernatant at 48 h and 72 h following transfection was collected and filtered through 0.45-μm PVDF membranes. The resulting virus was used to infect 293T, Hela and MCF7 cell lines (multiplicity of infection, 1:5) in the presence of 10 µg ml^−1^ polybrene (Santa Cruz, CAS 28728-55-4). Forty-eight hours following infection, cell sorting (AriaII, T105) was performed to select cells with similar fluorescence intensity for specific WT and mutant proteins.

#### Phosphoproteomics experiments and data analysis

Cell cultures were scraped, washed with PBS and frozen at –20 °C. Cells were lysed with 8 M urea, reduced and alkylated before overnight digestion with trypsin. Phosphorylated peptides were enriched with a Thermo High Select Phosphopeptide Enrichment Kit. Chromatography was performed using a Thermo EASY-nLC 1200 and a home-made 20-cm column packed with ReproSil-Pur, 120 Å, C18-AQ, 1.9-μm beads and a 110-min gradient. Data were acquired using a Thermo Orbitrap Lumo in data-dependent mode with a 2000V positive spray voltage, 300–1,600 *m/z* MS1 window, reseolution of 60,000 and maximum injection time of 50 ms. The cycle time was 3 s, with dynamic exclusion, charge state selection intensity and MIPS filters. MS2s were acquired with a isolation window of 0.7 *m/z*, HCD fragmentation at a fixed 33% collision energy, an Orbitrap resolution of 15,000 and auto maximum injection time using the Standard AGC Target.

For each target gene, the variant protein sequence was added to the UniProt reference database to generate a customized database. Fragpipe V22.0 (ref. ^63^) was then utilized for database searching, and the built-in phosphoproteome pipeline was adopted. Trypsin was selected as the digestion enzyme, and tryptic peptide lengths were set between 6 and 50 amino acids. Up to 2 missed cleavages were allowed per peptide, with a precursor ion tolerance of 10 ppm and a fragment ion tolerance of 20 ppm. Oxidation (+15.9949 Da) on Met and acetylation on the peptide N terminus (+42.0106 Da) were included as dynamic modifications; dynamic phosphorylation (+79.9663 Da) on Ser, Thr and Tyr was specified. Peptide identification was performed with a maximum FDR of 1% at the peptide level. Spectra were annotated using FragPipe-PDV (v1.2.0)^66^.

### Reporting summary

Further information on research design is available in the Nature Portfolio Reporting Summary linked to this article.

## Online content

Any methods, additional references, Nature Portfolio reporting summaries, source data, extended data, supplementary information, acknowledgements, peer review information; details of author contributions and competing interests; and statements of data and code availability are available at 10.1038/s41592-025-02797-x.

## Supplementary information

Reporting Summary

Supplementary Tables Supplementary Tables 1–6.

## Source data

Source Data Fig. 3 Statistical source data of Fig. 3i and j.

Source Data Fig. 4 Statistical source data of Fig. 4a–d.

Source Data Fig. 5 Statistical source data of Fig. 5a.

Source Data Fig. 6 Statistical source data of Fig. 6a.

## Data Availability

Information about 241 PTM-enriched MS/MS datasets used to build PTMAtlas is provided in Supplementary Table 1. PTM sites used for comparison were downloaded from public databases, including PhosphoSitePlus^13^ (downloaded 4 March 2020), UniProt^19^ (downloaded 14 February 2019), PLMD^20^ (downloaded 4 March 2020, v3.0) and N-GlycositeAtlas^21^ (downloaded 4 March 2020). The two SARS-CoV-2 phosphoproteomics datasets were downloaded from PRIDE^67^ under accession numbers PXD019113 and PXD018241, respectively. For the two CPTAC cohorts used for evaluation, somatic mutations were downloaded from the LinkedOmics database (https://www.linkedomics.org), and the raw MS/MS files for the six datasets from the two cohorts were downloaded from the Proteomic Data Commons (PDC, https://pdc.cancer.gov/pdc/). Pathogenic variants were obtained from ClinVar (https://www.ncbi.nlm.nih.gov/clinvar/, 28 January 2021). Somatic mutations were downloaded from TCGA data portals (https://portal.gdc.cancer.gov/, 12 January 2020). PTM sites identified from this study are available at http://deepmvp.ptmax.org. The MS proteomics data generated in this study have been deposited to the ProteomeXchange Consortium via the PRIDE^67^ partner repository with the dataset identifier PXD059468 at https://proteomecentral.proteomexchange.org/cgi/GetDataset?ID=PXD059468. Source data are provided with this paper.

## Code availability

The source code for DeepMVP is available at https://github.com/bzhanglab/DeepMVP. The specific version used in this study is archived at 10.5281/zenodo.15860222 (ref. ^68^).
