# Supplementary material for: DeepMVP: deep learning models trained on high-quality data accurately predict PTM sites and variant-induced alterations
Source: Nat Methods. 2025 Aug 26;22(9):1857–67. doi: 10.1038/s41592-025-02797-x (PMC12446062; doi:10.1038/s41592-025-02797-x)
Supplement: Supplementary file 1 — Reporting Summary [file 41592_2025_2797_MOESM1_ESM.pdf]

nature portfolio

Corresponding author(s): Bing Zhang

Last updated by author(s): 2025_05_08

Reporting Summary

Nature Portfolio wishes to improve the reproducibility of the work that we publish. This form provides structure for consistency and transparency in reporting. For further information on Nature Portfolio policies, see our Editorial Policies and the Editorial Policy Checklist.

Statistics

For all statistical analyses, confirm that the following items are present in the figure legend, table legend, main text, or Methods section.

n/a	Confirmed	
☐	☒	The exact sample size (*n*) for each experimental group/condition, given as a discrete number and unit of measurement
☒	☐	A statement on whether measurements were taken from distinct samples or whether the same sample was measured repeatedly
☒	☐	The statistical test(s) used AND whether they are one- or two-sided<br>*Only common tests should be described solely by name; describe more complex techniques in the Methods section.*
☒	☐	A description of all covariates tested
☒	☐	A description of any assumptions or corrections, such as tests of normality and adjustment for multiple comparisons
☐	☒	A full description of the statistical parameters including central tendency (e.g. means) or other basic estimates (e.g. regression coefficient) AND variation (e.g. standard deviation) or associated estimates of uncertainty (e.g. confidence intervals)
☒	☐	For null hypothesis testing, the test statistic (e.g. *F*, *t*, *r*) with confidence intervals, effect sizes, degrees of freedom and *P* value noted<br>*Give P values as exact values whenever suitable.*
☒	☐	For Bayesian analysis, information on the choice of priors and Markov chain Monte Carlo settings
☒	☐	For hierarchical and complex designs, identification of the appropriate level for tests and full reporting of outcomes
☒	☐	Estimates of effect sizes (e.g. Cohen's *d*, Pearson's *r*), indicating how they were calculated

Our web collection on statistics for biologists contains articles on many of the points above.

Software and code

Policy information about availability of computer code

Data collection	No software was used for data collection
Data analysis	All downloaded raw PTM MS/MS data were reanalyzed using MaxQuant (v1.6.5.0).<br>Global site level FDR across all the datasets was calculated using PGA (v1.0).<br>The deep learning models were implemented using Python with Tensorflow (v2.4.0, www.tensorflow.org).<br>Peptide similarity was evaluated using CD-HIT (v4.8.1)<br>The performance comparison of PTM site prediction were performed against eight published tools, including ModPred, MusiteDeep, GPS-MSP, GPS-SUMO, DeepPhos, NetPhos (v3.1), NetPhosPan, and UbiProber. While the performance comparison of variant impact prediction were performed against MusiteDeep, MIMP and VIPpred.<br>For the two CPTAC cohorts used for evaluation and cell line data generated in this study, FragPipe V22.0 pipelines of relevant proteomics platforms were then adopted for protein database searching using these customized databases to identify PTM sites from both reference and variant proteins. Spectra were annotated using FragPipe-PDV (v1.2.0). Germline variants were identified using HaplotypeCaller from the Sarek pipeline and annotated with Ensembl Variant Effect Predictor (v110.0) using the same gene annotation as for somatic mutations.<br>The annotation of pathogenic variants from ClinVar was performed with Ensembl Variant Effect Predictor (v102.0), while ANNOVAR (v20191024) was used for TCGA somatic mutation annotation.<br>The shapley value was computed using the function DeepExplainer from the python package shap (https://github.com/shap/shap, version 0.39.0).<br>The source code of DeepMVP is available at https://github.com/bzhanglab/DeepMVP.

For manuscripts utilizing custom algorithms or software that are central to the research but not yet described in published literature, software must be made available to editors and reviewers. We strongly encourage code deposition in a community repository (e.g. GitHub). See the Nature Portfolio guidelines for submitting code & software for further information.

Data

Policy information about availability of data

All manuscripts must include a data availability statement. This statement should provide the following information, where applicable:

- Accession codes, unique identifiers, or web links for publicly available datasets
- A description of any restrictions on data availability
- For clinical datasets or third party data, please ensure that the statement adheres to our policy

The information of 241 PTM enriched MS/MS datasets used to build PTMAtlas was provided in Supplementary Table 1. PTM sites used for comparison were downloaded from public databases, including PhosphoSitePlus (downloaded 03/04/2020), UniProt (downloaded 02/14/2019), PLMD (downloaded 03/04/2020, v3.0) and N-GlycositeAtlas (downloaded 03/04/2020). The two SARS-CoV-2 phosphoproteomics datasets were downloaded from PRIDE82 with accession number PXD019113 and PXD018241, respectively. For the two CPTAC cohorts used for evaluation, somatic mutations were downloaded from the LinkedOmics database (https://www.linkedomics.org), and the raw MS/MS files for the six datasets from the two cohorts were downloaded from the Proteomic Data Commons (PDC, https://pdc.cancer.gov/pdc/). Pathogenic variants were obtained from ClinVar (https://www.ncbi.nlm.nih.gov/clinvar/, 01/28/2021). Somatic mutations were downloaded from the data portals of TCGA (https://portal.gdc.cancer.gov/, 01/12/2020). PTM sites identified from this study are available at http://deepmvp.ptmax.org. The mass spectrometry proteomics data generated in this study have been deposited to the ProteomeXchange Consortium via the PRIDE82 partner repository with the dataset identifier PXD059468 at https://proteomecentral.proteomexchange.org/cgi/GetDataset?ID=PXD059468.

Human research participants

Policy information about studies involving human research participants and Sex and Gender in Research.

Reporting on sex and gender	N/A
Population characteristics	N/A
Recruitment	N/A
Ethics oversight	N/A

Note that full information on the approval of the study protocol must also be provided in the manuscript.

Field-specific reporting

Please select the one below that is the best fit for your research. If you are not sure, read the appropriate sections before making your selection.

☒ Life sciences ☐ Behavioural & social sciences ☐ Ecological, evolutionary & environmental sciences

For a reference copy of the document with all sections, see nature.com/documents/nr-reporting-summary-flat.pdf

Life sciences study design

All studies must disclose on these points even when the disclosure is negative.

Sample size	The sample size was determined based on previous publications and preliminary experiments to ensure adequate statistical power. No statistical method was used to predetermine sample size.
Data exclusions	Data points were excluded only in cases of technical failure (e.g., cell death unrelated to treatment, assay malfunction), and the exclusion criteria were pre-established. All exclusions are reported in the figure legends where applicable.
Replication	All experiments were independently repeated at least three times with consistent results. Biological replicates and technical replicates are specified in the figure legends.
Randomization	Samples were randomly assigned to experimental groups where applicable.
Blinding	Investigators were not blinded to group allocation during data collection or analysis unless otherwise specified. However, data quantification and statistical analysis were performed in an unbiased manner.

Reporting for specific materials, systems and methods

We require information from authors about some types of materials, experimental systems and methods used in many studies. Here, indicate whether each material, system or method listed is relevant to your study. If you are not sure if a list item applies to your research, read the appropriate section before selecting a response.

Materials & experimental systems

n/a	Involved in the study
☒	☐ Antibodies
☐	☒ Eukaryotic cell lines
☒	☐ Palaeontology and archaeology
☒	☐ Animals and other organisms
☒	☐ Clinical data
☒	☐ Dual use research of concern

Methods

n/a	Involved in the study
☒	☐ ChIP-seq
☒	☐ Flow cytometry
☒	☐ MRI-based neuroimaging

Eukaryotic cell lines

Policy information about cell lines and Sex and Gender in Research

Cell line source(s)	MCF7 cell line and 293T cell line from Prof. Yi Li's Lab (Baylor College of Medicine, TX, USA) were bought from ATCC. Hela cell line was from Molecular and Cellular Biology TC Core Lab (Baylor College of Medicine, TX, USA).
Authentication	Cell line identities were confirmed by short tandem repeat (STR) profiling, performed by ATCC, or Molecular and Cellular Biology TC Core Lab (Baylor College of Medicine, TX, USA). STR profiles were matched to reference profiles in established databases.
Mycoplasma contamination	All cell lines were tested regularly for mycoplasma contamination using MycoAlert™ Mycoplasma Detection Kit (Lonza)] and found to be free of mycoplasma during the course of the study.
Commonly misidentified lines (See ICLAC register)	None of the cell lines used in this study are listed in the ICLAC (International Cell Line Authentication Committee) database of misidentified cell lines. Alternatively, if any were previously misidentified but now authenticated, note it explicitly.
